# Contributions of the 5th Brazilian Congress on Health Policy, Planning, and Administration to the consolidation of the field of Public Health

**DOI:** 10.1590/0102-311XEN002225

**Published:** 2025-02-24

**Authors:** Carmem E. Leitão Araújo, Deivisson Vianna Dantas dos Santos, Isabela Soares Santos

**Affiliations:** 1 Faculdade de Medicina, Universidade Federal do Ceará, Fortaleza, Brasil.; 2 Departamento de Saúde Coletiva, Universidade Federal do Paraná, Curitiba, Brasil.; 3 Escola Nacional de Saúde Pública Sergio Arouca, Fundação Oswaldo Cruz, Rio de Janeiro, Brasil.

Since 2010, the Brazilian Public Health Association’s (ABRASCO, acronym in Portuguese) has held nationwide technical/scientific congresses on Health Policies, Planning, and Administration, one of the structuring cores of the Public Health field. Each edition of the Health Policies, Planning, and Administration congress provides lessons and experiences that enable progress in the field and subsequent congresses.

In November 2024, the 5th Brazilian Congress on Health Policy, Planning, and Administration (5th PPGS Congress) was conducted in Fortaleza. The northeastern capital, although characterized by a significant degree of social inequality, holds a deep history of struggles for the right to health and countless successful experiences that have contributed to the formation and consolidation of the Brazilian Health Reform Movement, as well as the constant construction of the Brazilian Unified National Health System (SUS, acronym in Portuguese). The event brought as a reflection: What are the contributions of the 5th PPGS Congress to Brazilian Public Health?

Public Health in Brazil stands out as an interdisciplinary field of knowledge, as well as a movement of unrestricted struggle for democracy and the SUS, being subjected to changes throughout its history and its role in Brazilian society ^1^. It is grounded in disciplinary fields or discursive formations that aim to reach the health-disease-care process as a social phenomenon in populations based on the perspectives of Epidemiology, Social and Human Sciences in Health, and Health Policies, Planning, and Administration ^2^.

Its consolidation process can be recognized by the existence, in 1996, of 24 academic Graduate Programs in the field of Public Health by the Brazilian Coordination for the Improvement of Higher Education Personnel (CAPES, acronym in Portuguese), expanding to 982 almost 30 years later. In addition, there is a strong investment in the establishment of multiprofessional residencies and undergraduate courses in Public Health, which has provided the opportunity to incorporate knowledge and practices in this field in the various professions in the healthcare field. Even so, only in 2025 the Health Policies, Planning, and Administration was recognized as a sub-area of Public Health by the Brazilian National Research Council (CNPq) ^3^.

The entire process of construction and development of the 5th PPGS Congress was conducted collectively and enabled an even broader vision, both of the creative power and challenges of this field, especially regarding the knowledge that has been produced. If, on the one hand, Public Health was founded by an integration between disciplines, discarding the mere multiprofessional juxtaposition ^4^, on the other hand, its expansion has led to a relative “subspecialization” from the perspectives brought by its disciplinary nuclei, which sometimes limits the necessary dialogue.

Public Health has been continually challenged by the present circumstances, especially the political-institutional, intersectional, socioeconomic, and environmental issues of the modern world. Health issues and the political situation are increasingly complex and require transdisciplinary actions. Despite this, the agendas of universities and research institutes have still been significantly impacted by the structural formation of Brazilian society. Since inequality is a central, fundamentally slavish and colonialist dimension, society has also been forged by a dense matrix of the coloniality of knowledge, with a predominance of Euro-North American-centric epistemology, despite the impact of Latin American critical thinking.

Public Health requires, in fact, the inclusion of increasingly critical and reflective perspectives aimed at formulating scientific questions that value the principles of democracy, human rights, equity, diversity, respect, tolerance, participation, and social control of society and sustainability of life, which permeate economic development linked to socio-environmental development. This strengthens the central claim of “*reducing inequalities, defending the right to health, and fighting for democracy and the emancipation of people, subjects, and our humanity*”, as Jairnilson Silva Paim ^5^ said at the closing of the 5th PPGS Congress.

In this sense, the subject defined for the congress encapsulated desires relating to *Policy, Knowledge, and Practices: Resistance and Insurgency in the confrontation of Health Inequities*. On the subject, there is a clear understanding that political, social, economic, and environmental choices and actions impact the health of the most vulnerable people and populations in a different way, especially for black women living at the periphery, Indigenous, Quilombolas and ribeirinhos communities, those with disabilities, older adults, transsexuals, living on the streets, LGBTQIA+, among others ^6^. In particular, it points to the persistence of brutal inequality across different regions of Brazil, including the conditions for the development of research, teaching, and community interfaces, the distribution of resources, services, and technologies has also been of growing concern ^7^.

The organization of the 5th PPGS Congress represented an exercise of intense dialogue for its composition. For example, traditional subjects of the field were associated with discussions on contemporary discussions on oppression of people, populations, and social groups. This proposal aimed at establishing an even greater dialogue in its foundation by incorporating various perspectives on the intersectionality of gender, race, social class, territorialities, and other categories that impact people’s lives and actions within the scope of SUS.

The context of life of vulnerable populations requires public policy responses that are based on equity, and, for this, it is necessary for Public Health to increasingly enable intersectional approaches, as reinforced throughout various activities of the 5th PPGS Congress. Most of the Brazilian population, which in turn holds the smallest power in Brazil, is subjected to processes that impact their health; thus, including their perspectives is fundamental when thinking about a fairer and less unequal society.

Thus, debates on policies, financing, healthcare networks, regionalization, social participation, training in work and health education, among other particular and recurrent subjects in the PPGS field, must recognize the perspectives and knowledge of all people and populations − including their struggle for legitimate social rights − as a vital part of academic knowledge, as well as of public administration and services. It is also a struggle for the universal right to health for the entire Brazilian population ^8^, and it is from this point of view that the concept of equity was treated during the Congress.

A strategy adopted to promote the inclusion of agendas and actors was to value the multiplicity of knowledge and invite all active Working Groups of ABRASCO to represent the Scientific Committee of the 5th PPGS Congress and, in this way, expand beyond the tradition.

There was an effort, in all stages of development, to include voices of various genders, ethnicities, generations, origins, and social classes, as well as people with various perspectives on each subject and contemporary discussions, who represented a potential contribution for the expansion of scientific knowledge ^9^.

It was recognized that, similar to the whole Brazilian society, the academic institutions of research and administration of the SUS reproduce power relations, oppressions, and vulnerabilities. Therefore, the decisions and leaders that organize such spaces must be increasingly critical, including knowledge and perspectives from various Brazilian populations that tend to be systematically invisible and have their rights neglected. These people need to be included in the debate on equity to achieve the universal right to health.

Thus, it was strategic, in the planning of the congress, to give visibility and value to the knowledge of Indigenous peoples and *Quilombolas*, understanding that they are vital to strengthen the perception that health and life are only possible if integrated with the environment, nature, and the joy of living. At the same time, the defended perspective differed from that prevailing in patriarchal capitalist societies, in which meritocracy, competition, domination, and hatred towards vulnerable people prevail along with the worsening of violence in general, especially against these populations.

Despite the most favorable circumstances for the consolidation of democracy and reconstruction of public health policies, global actions must not be disregarded, as well as the Brazilian political and economic elites aligned with the most conservative forces. Issues such as vaccination, abortion, socioeconomic sustainability, and decent work are regarded as catalysts for controversy by these conservative forces in the far-right politics, above all. Amid the use of various disinformation mechanisms, part of Brazilian society has been averse to science, history, institutions, and the structural changes necessary for a country that is sovereign and less unequal.

Thus, the Health Policies, Planning, and Administration dialogue was expanded with topics such as food insecurity, climate change, environmental racism, environmental sustainability, Indigenous people health, popular surveillance, public education, communication, disinformation, digital health, knowledge translation, scientific dissemination, popularization of science and promotion of public engagement, epistemicide, drugs, necropolitics, populations deprived of liberty, slave labor, migrations, autocracies and white supremacy, ultra-neoliberalism and the extreme right, institutional racism, misogyny, attacks on woman’s life due to unsafe abortions, rapes, and criminalization of abortion, among others.

The debates of the 5th PPGS Congress envisioned possible insurgent paths to the current crises, which, in addition to health, show several nuances of environmental/climatic origin, inequality, food insecurity, and dispute over land and water, resulting in large population migrations, worsening labor relationships, violence, and intensification of geopolitical disputes between the Global North and the rest of the world, generating wars and poverty ^10^.

In particular, the environmental and climate crises are consolidating themselves as increasingly necessary subjects in all current debates of the Health Policies, Planning, and Administration discussions. They highlight complex and growing challenges, with disasters and catastrophes, emergence and reemergence of epidemics, occurrence of pandemics, persistence of endemic diseases, as well as the expansion of poverty in a way that unveil the unsustainability of the global economic model and the increasing relevance of public action. Therefore, effective planning of the SUS is necessary, as well as effective policies and strategies against causes and agents of environmental and climate change. The State must provide the necessary social protection with a focus on addressing inequities and their impacts in a way that actually reaches all people who are unequally impacted.

National health systems, particularly in Brazil and those located in countries of the Global South, face numerous challenges to become truly equitable and more public at the same time that the global governance system reveals various weaknesses. Thus, the scientific knowledge of Health Policies, Planning, and Administration can be strategically mobilized to address these issues via citizen science.

The approach to these issues highlights the complexity of analyzing and conducting health policies and administration in the SUS, as well as the need of inducing research aimed at issues that demand greater articulation of knowledge between all cores of the field of Public Health ^11^. Complex issues require considering these intersections and discussions, as well as the associations between common elements and the materialization of a increasingly collective Health Policies, Planning, and Administration and Brazilian Congress of Public Health.

In this sense, the 5th PPGS Congress was a vital space for the ABRASCO Health Policies, Planning, and Administration Commission to advance in the formulation of its first Master Plan. As a pre-congress activity of the 5th PPGS Congress, the second workshop of the Master Plan was conducted, reinforcing that this first plan must be configured in a strategic document for Brazil. Four cores of action were structured: Teaching and Training in Health Policies, Planning, and Administration; Research, Theoretical/Methodological Development and Editorial Initiatives; and Public Health Policies and Social Movements. Echoes of the debates held in Fortaleza at the 5th PPGS Congress reached the 12th Brazilian Congress of Epidemiology, held in Rio de Janeiro in November 2024, when the Board of Directors of ABRASCO announced the construction of master plans for all cores of the field and, notably, decided that these movements be integrated to be presented at the 2025 Brazilian Congress of Public Health.

The 5th PPGS Congress also depicted ABRASCO’s closer relationship with the federal management of the SUS, based on the appreciation of Science. When Federal Administration resumes discussions on relevant public policies and evidence-based strategies and dialogues with the necessary criticisms on current political, institutional, and socioeconomic contexts, the debates undertaken regarding Brazilian Public Health contribute decisively to health policy and its agenda of reconstructing the SUS with equity in mind. While these discussions are conducted − of resuming the expansion of the family health strategy, revising the primary health care financing model, creating multidisciplinary teams (eMulti), and reorganizing the expanded and equitable training of healthcare professionals − there is a scenario of severe underfunding of the SUS, privatizations, increases in parliamentary amendments in mandatory federal expenditures, as well as permanent and new pressures on the Brazilian Government to disassociate and reduce the constitutional bases of health and education ^12^ ^,^ ^13^.

Over time, congresses have been points of contact between various actors, such as researchers, teachers, students, SUS administrators, professionals, and social movements, a fact that enables expanding the interfaces between the universes of thinking and acting, in order to contribute to the development of health policies, administration, and practices based on the highest quality science that considers local realities and socially constructed demands. In this way, 88 pre-congress activities were organized (51 workshops, 15 courses, 10 meetings, five forums, four meetings, two symposiums, and one seminar), four major debates, 52 round tables, and 2,089 approved papers distributed among 63 coordinated communication sessions, 130 conversation circles, and three artistic production sessions, in addition to the already traditional Paulo Freire Tent with six dialogue circles and four activities in related fields.

The Health Policies, Planning, and Administration congresses thus become open scientific meetings with an expanding space for exchanges, enabling the strengthening of the fight for universal right to health in a quality public system, as there are no rights without permanent mobilization. Thus, for this very reason, these congresses seek the reconstruction of equity in health as a founding principle of the SUS under a critical perspective. Providing spaces for meetings is an essential movement in Public Health, particularly after the experience and evidence brought during the COVID-19 pandemic.

Solutions to critical issues still largely lack after the Congress, including how to ensure a health system that considers social justice? How to face obstacles to the constitution of a truly universal health system that considers Brazilian heterogeneities? How to better encompass decolonial needs, possibilities, and epistemologies? And what can we prospect and act on collectively, considering the diversity and complexity of modern knowledge, epistemologies, and actors?

Aware of these complex challenges in Brazil and worldwide, resistance and insurgency in defense of life and an equitable SUS are demanded from social movements, the scientific community, Public Health, and Brazilian society. After a 5th PPGS Congress with this proposal, the importance of these ABRASCO meetings for the expansion of scientific knowledge and the agenda for the mobilization of Public Health is highlighted in defense of democracy and the SUS.
