# Supplementary material for: Contributions of the 5th Brazilian Congress on Health Policy, Planning, and Administration to the consolidation of the field of Public Health
Source: Cad Saude Publica. 2025 Feb 24;41(2):e00002225. doi: 10.1590/0102-311XEN002225 (PMC11863629; doi:10.1590/0102-311XEN002225)
Supplement: Translation

# Translation: Contribuições do 5º Congresso Brasileiro de Política, Planejamento e Gestão em Saúde para consolidação do campo da Saúde Coletiva

**DOI:** 10.1590/0102-311XPT002225

**Authors:** Carmem E. Leitão Araújo, Deivisson Vianna Dantas dos Santos, Isabela Soares Santos

**Affiliations:** 1 Faculdade de Medicina, Universidade Federal do Ceará, Fortaleza, Brasil.; 2 Departamento de Saúde Coletiva, Universidade Federal do Paraná, Curitiba, Brasil.; 3 Escola Nacional de Saúde Pública Sergio Arouca, Fundação Oswaldo Cruz, Rio de Janeiro, Brasil.

Desde 2010, a Associação Brasileira de Saúde Coletiva (Abrasco) realiza congressos técnico-científicos de âmbito nacional sobre Política, Planejamento e Gestão em Saúde, um dos eixos estruturantes do campo da Saúde Coletiva. A cada edição do congresso Política, Planejamento e Gestão em Saúde somam-se aprendizados e acúmulos que permitem avanços ao campo como um todo e aos congressos seguintes.

Em novembro de 2024, ocorreu, em Fortaleza, o 5º Congresso Brasileiro de Política, Planejamento e Gestão em Saúde (5º PPGS). A capital nordestina, apesar de marcada por considerável desigualdade social, tem uma rica história de lutas pelo direito à saúde e inúmeras experiências exitosas que vêm contribuindo para a conformação e consolidação do Movimento da Reforma Sanitária Brasileira, como também a construção cotidiana do Sistema Único de Saúde (SUS). O evento trouxe como reflexão: Quais as contribuições do 5º PPGS para a Saúde Coletiva brasileira?

A Saúde Coletiva no Brasil demarca-se como campo de conhecimento de natureza interdisciplinar, mas também como movimento de luta irrestrita pela democracia e pelo SUS, tomando corpo ao longo de sua história e do seu papel com relação à sociedade brasileira ^1^. Está fundamentada em núcleos disciplinares ou formações discursivas como eixos que buscam alcançar o fenômeno saúde-doença-cuidado enquanto processo social em populações a partir de perspectivas trazidas pela Epidemiologia, pelas Ciências Sociais e Humanas em Saúde e pela Política, Planejamento e Gestão em Saúde ^2^.

O seu processo de consolidação pode ser reconhecido pela existência, em 1996, de 24 programas de pós-graduação *stricto sensu* da área de Saúde Coletiva da Coordenação de Aperfeiçoamento de Pessoal de Nível Superior (CAPES), ampliando-se, quase trinta anos depois, para 982. Acresce-se o forte investimento na constituição de residências multiprofissionais e implantação de cursos de graduação em Saúde Coletiva, o que tem oportunizado a incorporação de conhecimentos e práticas desse campo nas diversas profissões da área da saúde. Ainda assim, apenas em 2025, o eixo de Política, Planejamento e Gestão em Saúde foi reconhecido como subárea da Saúde Coletiva pelo Conselho Nacional de Desenvolvimento Científico e Tecnológico (CNPq) ^3^.

Todo o processo de construção e desenvolvimento das atividades do 5º PPGS foi conduzido de forma coletiva e possibilitou o alcance de uma visão ainda mais ampla, tanto da potência criativa como dos desafios desse campo, principalmente em relação ao conhecimento que vem sendo produzido. Se por um lado, a Saúde Coletiva foi constituída tendo como um dos seus fundamentos a integração entre disciplinas, descartando a mera justaposição multiprofissional ^4^, por outro, a sua expansão levou a uma relativa “subespecialização” a partir das perspectivas trazidas por seus núcleos disciplinares, o que, por vezes, torna limitado o tão necessário diálogo.

A Saúde Coletiva tem sido continuamente provocada pela conjuntura em que se vive, principalmente pelas questões político-institucionais, interseccionais, socioeconômicas e ambientais da atualidade. Os problemas de saúde e a conjuntura política são cada vez mais complexos e exigem ações transdisciplinares. Apesar disso, as agendas das universidades e dos institutos de pesquisa ainda têm sofrido significativa influência da formação estrutural da sociedade brasileira. Por trazer em sua base fundante a desigualdade como dimensão central, fundamentalmente escravista e colonialista, a sociedade tem sido forjada também por uma densa matriz de colonialidade do saber, com predominância de epistemologia euronortecentrada, a despeito da incidência do pensamento crítico latino-americano.

A Saúde Coletiva requer, de fato, a inclusão de perspectivas cada vez mais crítico-reflexivas com vistas à formulação de questões científicas que valorizem os referenciais de democracia, direitos humanos, equidade, diversidade, respeito, tolerância, participação e controle social da sociedade e sustentabilidade da vida, que permeiem desenvolvimento econômico, atrelado ao socioambiental. Fortalece-se, assim, o pleito central de “*reduzir as desigualdades, de defender o direito à saúde, de brigar pela democracia e pela emancipação das pessoas, dos sujeitos e da nossa humanidade*”, como afirmou Jairnilson Silva Paim ^5^ no encerramento do 5º PPGS.

Nesta direção, o tema definido para o congresso condensou anseios: *Política, Saberes e Práticas: Resistência e Insurgência no Enfrentamento das Iniquidades em Saúde*. Nele há o nítido entendimento de que escolhas e ações políticas, sociais, econômicas e ambientais afetam de forma diferenciada a saúde das pessoas e populações mais vulnerabilizadas, especialmente mulheres, negras, periféricas, indígenas, quilombolas, ribeirinhas, com deficiência, idosas, trans, vivendo em situação de rua, LGBTQIA+, entre outras ^6^. Em particular, aponta para a persistência da brutal desigualdade entre as diferentes regiões do país, inclusive no que se remete às condições para o desenvolvimento de ações de pesquisa, ensino e interfaces junto à sociedade, distribuição de recursos, serviços e tecnologias também tem sido cada vez mais preocupante ^7^.

A organização do 5º PPGS representou um exercício de intenso diálogo para a sua composição. Por exemplo, temas tradicionais do campo foram articulados com as discussões sobre temáticas contemporâneas preocupadas com as opressões sobre pessoas, populações e grupos sociais. A proposta buscou ainda maior diálogo em suas composições com a incorporação de olhares para interseccionalidade de gênero, raça, classe social, territorialidades e outras categorias que incidem sobre a vida das pessoas e as ações no SUS.

O contexto de vida das populações vulnerabilizadas exige respostas refletidas em políticas públicas que tenham como base a equidade, e, para isso, faz-se necessário a Saúde Coletiva se abrir, cada vez mais, a abordagens interseccionais, como reforçado ao longo de várias atividades do 5º PPGS. A maioria da população, que reflete minoria com poder no Brasil, é afetada por processos vulnerabilizadores que repercutem na sua saúde, portanto a inclusão de suas perspectivas é condição ao se pensar em uma sociedade mais justa e menos desigual.

Portanto, os debates sobre políticas, financiamento, redes de atenção à saúde, regionalização, participação social, formação no trabalho e na educação na saúde, entre outros temas particulares e recorrentes ao campo Política, Planejamento e Gestão em Saúde, devem reconhecer as perspectivas e os conhecimentos dos movimentos e das lutas de pessoas e populações pela efetivação de direitos sociais legítimos como fundamentais para serem incluídos aos conhecimentos acadêmicos, da gestão e dos serviços. Trata-se de uma luta também pelo direito universal à saúde para toda a população brasileira ^8^, e é desse ponto de vista que o conceito de equidade foi tratado no Congresso.

Uma estratégia adotada para maior inclusão de pautas e atores/atrizes foi valorizar a multiplicidade de saberes e convidar todos os Grupos de Trabalho ativos da Abrasco para a indicação de uma representação para compor a Comissão Científica do 5º PPGS e, dessa forma, ampliar para além das tradicionais comissões do Política, Planejamento e Gestão em Saúde da Abrasco.

Houve um esforço, em todas as etapas de construção do Congresso, para inclusão da diversidade de pessoas debatedoras e palestrantes referente a questões de gênero, raça, geração, procedência regional e das respectivas instituições em relação à posição na sociedade, assim como a inclusão de pessoas com diferentes perspectivas sobre cada tema e de temas contemporâneos, que representassem potencial contribuição e acúmulo para ampliação do conhecimento científico ^9^.

Reconheceu-se que, da mesma forma, como toda a sociedade brasileira, as instituições acadêmicas de pesquisa e de gestão do SUS reproduzem relações de poder, opressões e vulnerabilizações, e, por isso, os movimentos e lideranças que organizam tais espaços devem ser cada vez mais críticos, incluindo conhecimentos e perspectivas de populações que no Brasil são sistematicamente invisibilizadas, têm seus direitos negligenciados e precisam ser incluídas no debate sobre qual equidade de fato se quer para alcance do direito universal à saúde.

Assim, foi estratégico, na construção do congresso, dar visibilidade e valorizar os saberes dos povos originários e quilombolas, entendendo que são fundamentais para fortalecer uma visão de mundo que sustenta que a saúde e a vida somente são possíveis se integradas ao ambiente, à natureza e à alegria de viver. Ao mesmo tempo, defendeu-se uma visão de mundo absolutamente diferente da vigente nas sociedades capitalistas patriarcais, na qual prevalecem a meritocracia, a competição, a dominação, o ódio às pessoas vulnerabilizadas com agravamento da violência em geral e, especificamente, contra essas populações.

Em que se pesem as circunstâncias mais propícias para a consolidação da democracia e reconstrução de políticas públicas de saúde, por outro, não são irrisórias as ações globais, mas sobretudo, das elites políticas e econômicas brasileiras alinhadas a forças mais conservadoras da sociedade. Temas como vacinação, aborto, sustentabilidade socioeconômica e trabalho digno são tomados como catalisadores de polêmicas por essas forças conservadoras na extrema-direita, sobretudo. Em meio à utilização de inúmeros mecanismos de desinformação, parcela da sociedade brasileira vem se mostrando avessa à ciência, à história, às instituições e às mudanças estruturais necessárias para um país que seja soberano e menos desigual.

Ampliou-se, assim, o diálogo da Política, Planejamento e Gestão em Saúde com temas como insegurança alimentar, mudanças climáticas e racismo ambiental, sustentabilidade ambiental, saúde indígena, vigilância popular, educação popular, comunicação, desinformação, saúde digital, translação do conhecimento, disseminação e divulgação científica, popularização da ciência e engajamento público, epistemicídio, drogas, necropolítica, população privada de liberdade, trabalho escravo, migrações, autocracias e supremacia branca, ultraneoliberalismo e extrema direita, racismo institucional, misoginia, ataque à vida das mulheres por abortos inseguros, estupros e criminalização por aborto, entre outros.

Os debates do 5º PPGS vislumbraram caminhos insurgentes possíveis para as crises atuais, as quais, para além de sanitária, têm diversas nuances de origem ambiental/climática, na desigualdade, na insegurança alimentar, na disputa pela terra e pela água, resultando em grandes migrações populacionais, na piora das relações de trabalho, na violência, no acirramento das disputas geopolíticas entre Norte Global e a maioria do mundo, gerando guerras e pobreza ^10^.

Em particular, as crises ambiental e climática se consolidam como temas cada vez mais necessários em todos os debates atuais do eixo de Política, Planejamento e Gestão em Saúde. Elas aguçam desafios complexos e crescentes, com desastres e catástrofes, emergência e reemergência de epidemias, ocorrência de pandemias e persistência de endemias, além de ampliação da pobreza, de modo a desvelar a insustentabilidade do modelo econômico global e a relevância cada vez maior da ação pública. São necessárias, portanto, o efetivo planejamento do SUS, assim como políticas e estratégias efetivas contra causas e causadores(as) das mudanças ambientais e climáticas. O Estado deve prover a necessária proteção social com foco no enfrentamento de iniquidades e de seus efeitos, isto é, que de fato chegue a todas as pessoas que são desigualmente afetadas.

Os sistemas nacionais de saúde, particularmente o brasileiro e aqueles situados no Sul Global, enfrentam inúmeros desafios para se tornarem realmente equitativos e mais públicos, ao passo que um sistema de governança global revela inúmeras fragilidades. Assim, o conhecimento científico próprio da Política, Planejamento e Gestão em Saúde pode ser mobilizado estrategicamente para essa incidência em uma perspectiva de ciência cidadã.

A abordagem desses assuntos mostra a complexidade de analisar e fazer política e gestão em saúde no SUS e também a necessidade de induzir pesquisas direcionadas para questões que demandam maior articulação de saberes entre todos os eixos do campo da Saúde Coletiva ^11^. Questões complexas exigem interseções e diálogo com outras formações discursivas do campo da Saúde Coletiva, inclusive pensando de forma articulada nos elementos em comum e na materialização da construção cada vez mais coletiva de seus eventos específicos, mas também do próprio Congresso Brasileiro de Saúde Coletiva (Abrascão).

Nessa direção, o 5º PPGS foi espaço fundamental para que a Comissão de Política, Planejamento e Gestão em Saúde da Abrasco avançasse em sua tarefa de formulação do seu primeiro Plano Diretor. Como atividade pré-congresso do 5º PPGS, a segunda oficina do Plano Diretor foi conduzida reforçando que esse primeiro plano deve se configurar em um documento estratégico para o país. Foram estruturados quatro eixos de atuação: Ensino e Formação em Política, Planejamento e Gestão em Saúde; Pesquisa, Desenvolvimento Teórico-metodológico e Iniciativas Editoriais; e Políticas Públicas de Saúde e Movimentos Sociais. Ecos dos debates realizados em Fortaleza no 5º PPGS alcançaram o 12º Congresso Brasileiro de Epidemiologia, no Rio de Janeiro em novembro de 2024, quando a Diretoria da Abrasco anunciou a construção de planos diretores para todos os eixos do campo, mas sobretudo, a decisão que esses movimentos sejam integrados para consolidação a ser apresentada durante o Abrascão 2025.

O 5º PPGS simbolizou também a maior aproximação da Abrasco com a Gestão Federal do SUS, pautada na valorização da Ciência. No momento em que a Gestão Federal retoma as discussões sobre políticas públicas relevantes e estratégias com base em evidências, e dialoga com as críticas necessárias frente aos contextos político institucional e socioeconômico atuais, os debates empreendidos à luz da tradição da Saúde Coletiva brasileira contribuem decisivamente para a política de saúde e a sua agenda de refundação do SUS com base na equidade. Enquanto se insere nesse debate, da retomada da expansão da estratégia de saúde da família, da revisão do modelo de financiamento da atenção primária à saúde (APS), da criação das equipes multiprofissionais (eMulti) e da reorganização da formação ampliada e equitativa de pessoas no SUS, tem-se um cenário de grave subfinanciamento do SUS, avanço das privatizações e aumento expressivo das emendas parlamentares em despesas federais obrigatórias, além das permanentes e renovadas pressões sobre o Governo Federal para desvincular e reduzir os pisos constitucionais da saúde e da educação ^12^ ^,^ ^13^.

Os congressos têm sido ao longo do tempo pontos de contato entre diferentes atores e atrizes, como pesquisadoras e pesquisadores, docentes, estudantes, gestoras e gestores do SUS, trabalhadoras e trabalhadores e movimentos sociais, fato que permite ampliar as interfaces entre os universos do pensar e do agir, de modo a contribuir para a construção de políticas de saúde e práticas de gestão e trabalho em saúde pautadas na ciência da mais alta qualidade, com sensibilidade às realidades locais e às demandas socialmente construídas. Dessa forma, foram organizadas 88 atividades pré-congresso (51 oficinas, 15 cursos, 10 reuniões, cinco fóruns, quatro encontros, dois simpósios e um seminário), quatro grandes debates, 52 mesas redondas e 2.089 trabalhos aprovados distribuídos entre 63 sessões de comunicações coordenadas, 130 rodas de conversa e três sessões de produção artística, além da já tradicional Tenda Paulo Freire com seis rodas de diálogo e quatro vivências em territórios.

Os congressos de Política, Planejamento e Gestão em Saúde se tornam, assim, encontros científicos plurais com cada vez mais espaços de trocas, de fortalecimento de luta pelo direito universal à saúde, de um sistema público e gratuito de qualidade, uma vez que não há direitos sem mobilização permanente e, por isso mesmo, na reconstrução da equidade em saúde como princípio fundante do SUS em perspectiva crítica. Oportunizar espaços para encontros é um movimento essencial na Saúde Coletiva, particularmente após a experiência e as evidências trazidas no contexto da pandemia por COVID-19.

Em grande medida, permanecem buscas de soluções para problemas críticos após o Congresso: como garantir um sistema de saúde com justiça social? Como enfrentar obstáculos para constituição de um sistema de saúde de fato universal e que considere as heterogeneidades brasileiras? Como olhar cada vez mais para necessidades, possibilidades e epistemologias decoloniais? E o que podemos prospectar e agir conjuntamente, considerando a diversidade de saberes, epistemologias e atores/atrizes com representatividade devido à complexidade do tempo presente?

Tendo consciência desses complexos desafios no Brasil e no mundo, é exigido, tanto de movimentos sociais quanto da comunidade científica, da Saúde Coletiva e da sociedade brasileira, resistência e insurgências em defesa da vida e de um SUS equânime. Após um 5º PPGS com essa proposta, reitera-se a importância desses encontros da Abrasco para o acúmulo de conhecimento científico e a agenda à mobilização da Saúde Coletiva, em defesa da democracia e do SUS.

# Translation: Contribuciones del 5.º Congreso Brasileño de Política, Planificación y Gestión de la Salud a la consolidación del campo de la Salud Colectiva

**DOI:** 10.1590/0102-311XES002225

**Authors:** Carmem E. Leitão Araújo, Deivisson Vianna Dantas dos Santos, Isabela Soares Santos

**Affiliations:** 1 Faculdade de Medicina, Universidade Federal do Ceará, Fortaleza, Brasil.; 2 Departamento de Saúde Coletiva, Universidade Federal do Paraná, Curitiba, Brasil.; 3 Escola Nacional de Saúde Pública Sergio Arouca, Fundação Oswaldo Cruz, Rio de Janeiro, Brasil.

Desde 2010, la Asociación Brasileña de Salud Colectiva (ABRASCO) realiza congresos técnico-científicos en ámbito nacional sobre Política, Planificación y Gestión de la Salud, uno de los ejes estructurantes del campo de la Salud Colectiva. A cada edición del Congreso Política, Planificación y Gestión de la Salud se suman aprendizajes que permiten avances en el campo y en los congresos siguientes.

En noviembre de 2024 ocurrió en Fortaleza el 5.º Congreso Brasileño de Política, Planificación y Gestión de la Salud (5.º PPGS). Una de las capitales del Nordeste brasileño, a pesar de estar marcada por una fuerte desigualdad social, Fortaleza tiene una rica historia de luchas por el derecho a la salud y numerosas experiencias exitosas que han contribuido a la conformación y consolidación del Movimiento de Reforma Sanitaria Brasileña, así como a la construcción cotidiana del Sistema Único de Salud brasileño (SUS). El evento trajo como reflexión: ¿Cuáles son las contribuciones del 5.º PPGS a la salud pública brasileña?

La Salud Colectiva en Brasil se destaca como un campo de conocimiento de carácter interdisciplinario, pero también como un movimiento de lucha irrestricta por la democracia y el SUS, que se va configurando a lo largo de su historia y de acuerdo con su papel en relación con la sociedad brasileña ^1^. Está basada en núcleos disciplinarios o formaciones discursivas como ejes que buscan alcanzar el fenómeno salud-enfermedad-atención como proceso social en las poblaciones desde perspectivas aportadas por la epidemiología, las ciencias sociales y humanas en salud y por el Política, Planificación y Gestión de la Salud ^2^.

Su proceso de consolidación puede ser reconocido por la existencia, en 1996, de 24 programas de posgrado en Salud Colectiva de la Coordinación de Perfeccionamiento del Personal de Nivel Superior (CAPES), ampliándose, casi treinta años después, a 982. Además, existe una fuerte inversión en el establecimiento de residencias multiprofesionales y en la implementación de cursos de grado en Salud Colectiva, lo que ha brindado la oportunidad de incorporar conocimientos y prácticas de este campo en las diversas profesiones del área de la salud. Aun así, apenas en 2025, el eje Política, Planificación y Gestión de la Salud fue reconocido como una subárea de la Salud Colectiva por el Consejo Nacional de Desarrollo Científico y Tecnológico (CNPq) ^3^.

Todo el proceso de construcción y desarrollo de las actividades del 5.º PPGS se llevó a cabo de manera colectiva y permitió alcanzar una visión aún más amplia, tanto del poder creativo como de los desafíos de este campo, especialmente en relación con el conocimiento que se ha producido. Si, por un lado, la Salud Colectiva se constituyó con la integración entre disciplinas como uno de sus fundamentos descartando la mera yuxtaposición multiprofesional ^4^, por otro lado, su expansión ha llevado a una relativa “subespecialización” desde las perspectivas aportadas por sus núcleos disciplinarios, lo que a veces limita el diálogo necesario.

La Salud Colectiva ha sido provocada continuamente por la coyuntura en que vivimos, principalmente por las problemáticas político-institucionales, interseccionales, socioeconómicas y ambientales de la actualidad. Los problemas de salud y la situación política son cada vez más complejos y requieren acciones transdisciplinarias. A pesar de ello, las agendas de las universidades e institutos de investigación han sido significativamente influenciadas por la formación estructural de la sociedad brasileña. Por tener en su base fundacional la desigualdad como dimensión central, fundamentalmente esclavista y colonialista, la sociedad también se ha forjado a partir de una densa matriz de colonialidad del conocimiento, con predominio de la epistemología eurocéntrica, a pesar de la incidencia del pensamiento crítico latinoamericano.

La Salud Colectiva requiere, de hecho, la inclusión de perspectivas cada vez más crítico-reflexivas con el objetivo de formular preguntas científicas que valoren los referentes de la democracia, los derechos humanos, la equidad, la diversidad, el respeto, la tolerancia, la participación y el control social de la sociedad y la sostenibilidad de la vida, que permean el desarrollo económico, vinculado al socioambiental. De esta manera, se fortalece la reivindicación central de “*reducir las desigualdades, defender el derecho a la salud, luchar por la democracia y la emancipación de las personas, de los sujetos y de nuestra humanidad*”, tal como lo afirmó Jairnilson Silva Paim ^5^ en el cierre del 5.º PPGS.

En este sentido, el tema definido para el Congreso condensó los anhelos: políticas, conocimientos y prácticas: la resistencia e insurgencia en el enfrentamiento de las iniquidades en la salud. Sobre el tema, se entiende claramente que las decisiones y acciones políticas, sociales, económicas y ambientales afectan de manera diferente la salud de las personas y poblaciones más vulnerables, especialmente las mujeres, negras, periféricas, indígenas, quilombolas, ribereñas, con discapacidad, los adultos mayores, trans, en situación de calle, LGBTQIA+, entre outras ^6^. En particular, señala la persistencia de una desigualdad brutal entre las diferentes regiones del país, incluso respecto a las condiciones para el desarrollo de las acciones de investigación, enseñanza e interfaz con la sociedad; la distribución de recursos, servicios y tecnologías también ha sido cada vez más preocupante ^7^.

La organización del 5.º PPGS representó un ejercicio de diálogo intenso para su composición. Por ejemplo, los temas tradicionales del campo se articularon con debates sobre temas contemporáneos relacionados con la opresión de las personas, las poblaciones y los grupos sociales. La propuesta buscó un diálogo aún mayor en sus composiciones con la incorporación de visiones sobre la interseccionalidad de género, raza, clase social, territorialidades y otras categorías que afectan la vida y las acciones de las personas en el Sistema Único de Salud (SUS).

El contexto de vida de las poblaciones vulnerables requiere respuestas reflejadas en políticas públicas basadas en la equidad y, para ello, es necesario que la Salud Colectiva se abra cada vez más a enfoques interseccionales, como se refuerza a lo largo de diversas actividades del 5.º PPGS. La mayoría de la población, que refleja una minoría con poder en Brasil, se ve afectada por procesos que vulnerabilizan y repercuten en su salud, por lo que la inclusión de sus perspectivas es una condición a la hora de pensar en una sociedad más justa y menos desigual.

Por lo tanto, los debates sobre políticas, financiamiento, redes de atención a la salud, regionalización, participación social, formación profesional y en educación para la salud, entre otros temas particulares y recurrentes en el campo del Política, Planificación y Gestión de la Salud, deben reconocer las perspectivas y los conocimientos de los movimientos y luchas de las personas y las poblaciones por la realización de los derechos sociales legítimos como fundamentales para ser incluidos en el conocimiento académico, de la gestión y los servicios. Es también una lucha por el derecho universal a la salud para toda la población brasileña ^8^, y es desde este punto de vista que se trató el concepto de equidad en el Congreso.

Una estrategia adoptada para una mayor inclusión de agendas y actores/actrices fue valorar la multiplicidad de conocimientos e invitar a todos los grupos de trabajo activos de Abrasco a nominar una representación para componer el Comité Científico del 5.º PPGS y, de esta manera, expandirse más allá de los comités tradicionales del Política, Planificación y Gestión de la Salud de ABRASCO.

Hubo un esfuerzo, en todas las etapas de la construcción del Congreso, por incluir la diversidad de debatientes y ponentes en temas de género, raza, generación, origen regional y las instituciones respectivas en relación con su posición en la sociedad, así como la inclusión de personas con diferentes perspectivas sobre cada tema y temas contemporáneos, que representaban un potencial aporte y acumulación para la expansión del conocimiento científico ^9^.

Se reconoció que, de la misma manera, al igual que toda la sociedad brasileña, las instituciones académicas de investigación y gestión del SUS reproducen las relaciones de poder, las opresiones y las vulnerabilidades, y, por lo tanto, los movimientos y líderes que organizan esos espacios deben ser cada vez más críticos, incluyendo el conocimiento y las perspectivas de las poblaciones que son sistemáticamente invisibilizadas en Brasil, tienen sus derechos desatendidos y deben ser incluidas en el debate sobre qué tipo de equidad es necesaria para lograr el derecho universal a la salud.

Así, fue estratégico, en la construcción del Congreso, dar visibilidad y valor a los saberes de los pueblos originarios y quilombolas, entendiendo que son fundamentales para fortalecer una visión del mundo que sostiene que la salud y la vida solo son posibles si se integran con el medio ambiente, la naturaleza y la alegría de vivir. Al mismo tiempo, se defendió una visión del mundo absolutamente diferente a la que prevalece en las sociedades capitalistas patriarcales, en las que predominan la meritocracia, la competencia, la dominación y el odio a las personas vulnerables con el recrudecimiento de la violencia en general y, específicamente, contra estas poblaciones.

A pesar de las circunstancias más favorables para la consolidación de la democracia y la reconstrucción de las políticas públicas de salud, en cambio, las acciones globales no son irrisorias, sobre todo las de las élites políticas y económicas brasileñas alineadas con las fuerzas más conservadoras de la sociedad. Temas como la vacunación, el aborto, la sostenibilidad socioeconómica y el trabajo decente son tomados como catalizadores de la controversia por estas fuerzas conservadoras de la extrema derecha. En medio del uso de numerosos mecanismos de desinformación, parte de la sociedad brasileña ha sido reacia a la ciencia, la historia, las instituciones y los cambios estructurales necesarios para un país soberano y menos desigual.

Así, el diálogo del Política, Planificación y Gestión de la Salud se amplió con temas como la inseguridad alimentaria, el cambio climático y el racismo ambiental, la sostenibilidad ambiental, la salud indígena, la vigilancia en salud pública, la educación popular, la comunicación, la desinformación, la salud digital, la traducción del conocimiento, la difusión y divulgación científica, la popularización de la ciencia y la participación pública, el epistemicidio, las drogas, la necropolítica, la población privada de libertad, el trabajo esclavo, las migraciones, las autocracias y la supremacía blanca, el ultraneoliberalismo y la extrema derecha, el racismo institucional, la misoginia, los ataques a la vida de las mujeres a través de abortos inseguros, las violaciones y la penalización del aborto, entre otros.

Los debates del 5.º PPGS vislumbraron posibles caminos insurgentes hacia las crisis actuales, que, además de la salud, tienen varios matices de origen ambiental/climático, en la desigualdad, la inseguridad alimentaria, la disputa por la tierra y el agua, lo que resulta en grandes migraciones de población, el empeoramiento de las relaciones laborales, la violencia y la intensificación de las disputas geopolíticas entre el Norte Global y la mayor parte del mundo, generando las guerras y la pobreza ^10^.

En particular, las crisis ambiental y climática se están consolidando como temas cada vez más necesarios en todos los debates actuales del eje Política, Planificación y Gestión de la Salud. Ellas exacerban desafíos complejos y crecientes con desastres y catástrofes, la aparición y reaparición de epidemias, la emergencia de pandemias y la persistencia de enfermedades endémicas, así como la expansión de la pobreza, con el fin de develar la insostenibilidad del modelo económico mundial y la creciente relevancia de la acción pública. Por lo tanto, es necesaria una planificación efectiva del SUS, así como políticas y estrategias efectivas contra las causas y los y las causantes del cambio ambiental y climático. El Estado debe brindar la protección social necesaria con un enfoque en enfrentar las inequidades y sus efectos, es decir, que realmente llegue a todas las personas que se ven afectadas de manera desigual.

Los sistemas nacionales de salud, en particular el brasileño y los ubicados en el Sur Global, enfrentan numerosos desafíos para ser verdaderamente equitativos y más públicos, mientras que un sistema de gobernanza global revela numerosas debilidades. Así, el conocimiento científico del Política, Planificación y Gestión de la Salud puede ser movilizado estratégicamente para esta incidencia en una perspectiva de ciencia ciudadana.

El abordaje de estas cuestiones muestra la complejidad de analizar y hacer política y gestión de salud en el SUS, así como la necesidad de inducir investigaciones dirigidas a cuestiones que demandan una mayor articulación del conocimiento entre todos los ejes del campo de la Salud Colectiva ^11^. Cuestiones complejas requieren intersecciones y diálogos con otras formaciones discursivas en el campo de la Salud Colectiva, incluyendo el pensamiento de forma articulada sobre los elementos comunes y la materialización de la construcción cada vez más colectiva de sus eventos específicos, pero también del propio Congreso Brasileño de Salud Colectiva (Abrascão).

En este sentido, el 5.º PPGS fue un espacio fundamental para que la Comisión Política, Planificación y Gestión de la Salud de ABRASCO avanzara en su tarea de formular su primer Plan Director. Como actividad precongreso del 5.º PPGS, se realizó el segundo taller del Plan Director, reforzando que este primer plan debe configurarse en un documento estratégico para el país. Se estructuraron cuatro ejes de actuación: enseñanza y capacitación en Política, Planificación y Gestión de la Salud; investigación, desarrollo teórico-metodológico e iniciativas editoriales; y políticas de salud pública y movimientos sociales. Ecos de los debates realizados en Fortaleza en el 5.º PPGS llegaron al 12.º Congreso Brasileño de Epidemiología, en Río de Janeiro en noviembre de 2024, cuando el Consejo de Administración de Abrasco anunció la construcción de Planes Directores para todos los ejes del campo, pero sobre todo, la decisión de que estos movimientos se integren para su consolidación que se presentará durante el Abrascão 2025.

El 5.º PPGS también simbolizó la relación más estrecha de Abrasco con la gestión federal del SUS, basada en la valoración de la Ciencia. En un momento en que la Administración Federal retoma las discusiones sobre las políticas públicas relevantes y las estrategias basadas en la evidencia, y dialoga con las críticas necesarias frente a los contextos políticos, institucionales y socioeconómicos actuales; los debates emprendidos a la luz de la tradición de la Salud Colectiva brasileña contribuyen decisivamente a la política de salud y a su agenda de refundación del SUS basada en la equidad. Mientras se establece este debate, la reanudación de la expansión de la estrategia de salud de la familia, la revisión del modelo de financiación de la atención primaria de salud (APS), la creación de equipos multiprofesionales (eMulti) y la reorganización de la formación ampliada y equitativa de las personas en el SUS, se presenta un escenario de grave desfinanciación del SUS, el avance de las privatizaciones y un aumento significativo de las enmiendas parlamentarias tratando de los gastos federales obligatorios, además de las permanentes y renovadas presiones al Gobierno Federal para desvincular y reducir los pisos constitucionales de la salud y la educación ^12^ ^,^ ^13^.

A lo largo del tiempo, los congresos han sido puntos de contacto entre diferentes actores, como los investigadores, los docentes, los estudiantes, los gestores del SUS, los trabajadores y los movimientos sociales, hecho que permite ampliar las interfaces entre los universos de pensar y actuar, con el fin de contribuir a la construcción de políticas de salud y de prácticas de gestión y trabajo en salud basadas en la ciencia de la más alta calidad con sensibilidad a las realidades locales y a las demandas socialmente construidas. De esta manera, se organizaron 88 actividades precongreso (51 talleres, 15 cursos, 10 encuentros, cinco foros, cuatro encuentros, dos simposios y un seminario), cuatro grandes debates, 52 mesas redondas y 2.089 trabajos aprobados distribuidos en 63 sesiones coordinadas de comunicación, 130 círculos de conversación y tres sesiones de producción artística, además de la ya tradicional Carpa Paulo Freire con seis círculos de diálogo y cuatro experiencias en territorios.

Los congresos del Política, Planificación y Gestión de la Salud se convierten así en encuentros científicos plurales con cada vez más espacios de intercambio, de fortalecimiento de la lucha por el derecho universal a la salud, por un sistema de calidad público y gratuito, ya que no hay derechos sin movilización permanente y, por eso, se busca la reconstrucción de la equidad en salud como principio fundacional del SUS en una perspectiva crítica. Brindar espacios de encuentro es un movimiento esencial para la Salud Colectiva, particularmente después de la experiencia y la evidencia aportadas en el contexto de la pandemia de la COVID-19.

En gran medida, después del Congreso aún se buscan soluciones a problemas críticos: ¿Cómo garantizar un sistema de salud con justicia social? ¿Cómo enfrentar los obstáculos para la constitución de un sistema de salud verdaderamente universal que considere las heterogeneidades brasileñas? ¿Cómo considerar cada vez más las necesidades, posibilidades y epistemologías decoloniales? ¿Y qué podemos prospectar y actuar juntos, considerando la diversidad de conocimientos, epistemologías y actores/actrices con representatividad debido a la complejidad del tiempo presente?

Conscientes de estos complejos desafíos en Brasil y en el mundo, se requieren resistencias e insurgencias en defensa de la vida y de un SUS equitativo tanto de los movimientos sociales como de la comunidad científica, de la Salud Colectiva y de la sociedad brasileña. Luego de un 5.º PPGS con esta propuesta, se reitera la importancia de estos encuentros de Abrasco para la acumulación de conocimiento científico y la agenda para la movilización de la Salud Colectiva, en defensa de la democracia y del SUS.
